# Diabetes in Danish Bank Voles (M. glareolus): Survivorship, Influence on Weight, and Evaluation of Polydipsia as a Screening Tool for Hyperglycaemia

**DOI:** 10.1371/journal.pone.0022893

**Published:** 2011-08-04

**Authors:** Bryan Schønecker, Tonny Freimanis, Irene Vejgaard Sørensen

**Affiliations:** 1 Department of Biology, University of Copenhagen, Copenhagen, Denmark; 2 Department of Veterinary Disease Biology, University of Copenhagen, Copenhagen, Denmark; Institut Pluridisciplinaire Hubert Curien, France

## Abstract

**Background:**

Previous studies have concluded that the development of polydipsia (PD, a daily water intake ≥21 ml) among captive Danish bank voles, is associated with the development of a type 1 diabetes (T1D), based on findings of hyperglycaemia, glucosuria, ketonuria/-emia, lipemia, destroyed beta cells, and presence of autoantibodies against GAD65, IA-2, and insulin.

**Aim and Methods:**

We retrospectively analysed data from two separate colonies of Danish bank voles in order to 1) estimate survivorship after onset of PD, 2) evaluate whether the weight of PD voles differed from non-PD voles, and, 3), evaluate a state of PD as a practical and non-invasive tool to screen for voles with a high probability of hypeglycaemia. In addition, we discuss regional differences related to the development of diabetes in Scandinavian bank voles and the relevance of the Ljungan virus as proposed etiological agent.

**Results:**

We found that median survival after onset of PD is at least 91 days (lower/upper quartiles = 57/134 days) with a maximum recording of at least 404 days survivorship. The development of PD did not influence the weight of Danish bank voles. The measures of accuracy when using PD as predictor of hyperglycaemia, i.e. sensitivity, specificity, positive predictive value, and negative predictive value, equalled 69%, 97%, 89%, and 89%, respectively.

**Conclusion:**

The relatively long survival of Danish PD bank voles suggests potentials for this model in future studies of the long-term complications of diabetes, of which some observations are mentioned. Data also indicates that diabetes in Danish bank is not associated with a higher body weight. Finally, the method of using measurements of daily water intake to screen for voles with a high probability of hyperglycaemia constitutes a considerable refinement when compared to the usual, invasive, methods.

## Introduction

Diabetes has been recognized for millennia's [1] and is characterized by persistent hyperglycaemia due to defects in either insulin secretion, insulin action, or a combination [2]. Its classical symptoms includes polydipsia (PD), glucosuria, polyuria, unexplained weight loss, and sometimes coma [3]. Diabetes can damage any organs in the body [4], and even the most well-controlled patients still experience serious long-term complications [5]. The main complications of diabetes are caused by development of retinopathy, nephropathy, neuropathy and circulatory dysfunctions, leading to impaired vision/blindness, renal failure, amputations, and cardiovascular diseases, respectively [1], [3], [6], [7]. Other complications could e.g. be various rheumatic diseases [8], weakening of the skeletal muscles [9], problems relating to intestinal microbial overgrowth [10], [11], gastrointestinal dysfunctions [12], susceptibility towards development of cancer [13], infertility [14], and a notably decreased life-expectancy [15]–[18].

Approximately 5–10% of the diabetic patients suffers from type 1 diabetes (T1D) [19], which show a peak incidence in childhood [20] but can develop at any age [21], [22]. The incidence of T1D does not seem to show gender differences in the ages below 15, but present a male/female ratio of 1.5–1.8 in adulthood [23], [24]. Human T1D is associated with a chronic autoimmune process for which the primary cause remains unknown [25], [26], and this so-called insulitis slowly destroys the insulin-producing beta cells in the islets of Langerhans and probably also results in a reduced function of the remaining islets [25], [27]. Besides insulitis, which has been shown to be present in 70% of newly diagnosed T1D patients [28], T1D has also for long been associated with presence of autoantibodies against islet cells (ICA), insulin (IAA), the 65 kDa isoform of glutamic acid decarboxylase (GAD65), and the two tyrosine phosphatase proteins ICA512 (IA-2) and Phogrin [2]. Presence of autoantibodies can precede T1D by many years but so far no evidence has been produced that they play an active role in the pathogenesis of T1D [29]. Experiments will typically, and for obvious reasons, have to make use of animal models, and several spontaneous models are in fact available. The two most widely used models of T1D are at present the nonobese diabetic (NOD) mouse [30] and the BioBreeding diabetes-prone (BB-DP) rat [31].

The prevalence of T1D in NOD mice shows a marked sex-difference (70–90% affected individuals among females; 20–65% among males), with a typical onset between the age of 3–6 months [32]. T1D is accompanied by the presence of autoantibodies against GAD65, IAA, IA-2, but the true nature of these autoantibodies has been questioned [33]. Also accompanying the development of T1D in NOD mice is an insulitis of a different and more aggressive type than what is observed in human T1D where insulitis is mild [34], [35], [36]. Insulitis in NOD mice succeeds a long period with benign peri-insulitis, which is seen in practically every NOD mouse but not observed in human T1D [37]–[39].

In contrast to NOD mice, BB-DP rats do not show any sex bias regarding proneness to develop T1D (prevalence: 50–80% in both sexes [32]), and their accompanying insulitis appears similar to what is observed in human T1D [40]. The development of T1D in the BB-DP rat typically shows an abrupt onset at ages 60–120 days [41] with a mean of 90 days in most publications; is accompanied by autoantibodies against ICA, whereas presence of autoantibodies against GAD and IAA is, however, controversial. [40]. Diabetic BB-DP rats usually die within 2 weeks of ketoacidosis [42] unless they are treated with insulin, whereas NOD mice are able to survive for considerably longer without treatment (1–12 weeks [39], [43]).

Obviously, both NOD and BB-DP models have their particular limitations, and the same can be said about other recognized models, e.g. the Long-Evans Tokushima Lean (LETL) rat [44], the Komeda diabetes-prone (KDP) rat [45], and the LEW.1AR1/Ztm-iddm (LEW) rat [46], [47]. It should also be noted that there is no clear line separating animal models for T1D from animal models for type 2 diabetes [42].

The use of captive bank voles (Myodes glareolus) has only recently been proposed as a suitable model for diabetes research. Observations of PD among a large fraction of captive Danish bank voles were first published in 1986 [48]. PD was at the time believed to be some sort of maladjusted response to captivity, and PD was further found to be associated with highly increased mortalities. PD is also, as mentioned above, one of the classical signs of diabetes, since a state of hyperglycaemia will transgress the renal threshold (approx. 10 mM in humans; not determined in voles), leading to glucosuria, polyuria, and dehydration, which in the end triggers an impulse to drink excessively, i.e. PD [49], [50]. Observations made by the authors in the late nineties likewise associated the frequent development of PD among Danish bank voles with highly increased mortalities, in addition to the development of supposed lumps/tumours, eye problems, fluffy fur, occasional loss of fur in the groin area, changes in gait (walking on heels as opposed to normal toe-walk), weight losses, lack of diurnal rhythm in activity, reduced fertility, and, in some cases, development of large volumes of intestinal gas. In addition, PD voles were tested positive for glucosuria, hyperglycaemia, and lipemia, thus demonstrating their potential uses as a new animal model for diabetes [51], [52]. Onset is early (approx. two months) and males develop PD roughly three times as often as females when housed in isolation without additional stressors (30–34% vs. 11–13%; [51]). However, exposure to pre-weaning stress increased or decreased the incidence of PD in both genders to 53% and 13%, respectively, dependant on the method used [52]. A follow-up study associated PD in captive Danish bank voles with the presence of ketonuria, ketonemia, hyperlipidemia, a major loss of insulin-positive beta cells, and autoantibodies against the same three markers (GAD65, IA-2, IAA) commonly used to predict human T1D. The study concluded that captive Danish bank voles could develop a diabetes consistent with a diagnose of T1D [53]. Insulitis was, however, rarely observed in T1D Danish bank voles which presents a striking difference to other T1D models, such as NOD mice [54], BB-DP rats [55], KDP rats [45], and LETL rats [44].

The development of diabetes in bank voles has only been subject to a very few studies compared to the e.g. approx. 3000 papers describing diabetes in NOD and BB-DP models. Three papers address Danish bank voles [51]–[53] and five papers address Swedish bank voles [56]–[60], but nevertheless, available evidence suggests that the development of diabetes in Scandinavian bank voles are subject to regional differences regarding aspects such as predominant type, incidence, and proneness to develop capture-induced hyperglycaemia (see discussion).

The aim of this study was to provide an actual quantification of the relatively long survival after onset of PD in Danish bank voles (as briefly mentioned in reference [52]). Second, we intend to use available data to analyse whether established PDs present a weight loss compared to non-PDs (as briefly mentioned in reference [53]), and thirdly, we intend to evaluate the accuracy of an easy and non-invasive method to screen for voles with a high probability of also being diabetic. The method consists of measuring average daily water intake (DWI) to establish a state of PD, which in turn is one of the consequences of a state of hyperglycaemia, i.e. diabetes.

## Materials and Methods

### General approach

This study is based on a retrospective analysis of data from two separate colonies of Danish bank voles, founded and housed at the University of Copenhagen in 1995–1997 and 2000–2003. Animal care and animal use conformed to institutional policies and guidelines and were in accordance with the International Guiding Principles for Biomedical Research Involving Animals (1985) and the ethical guidelines proposed by ISAE Ethics Committee (2002).

### Animals, housing, and weaning

The founders of the two colonies were caught on the Danish island Zealand in 1995 and 2000 using live traps (“Ugglan special No1” made by the Swedish company Grahnab AB: http://www.grahnab.se). Capture index (number of animals caught during 100 “trap-nights”) for the first colony was not recorded while it was 6.78 (189 bank voles caught using 2789 trap-nights) for the second colony, from which some of the wild caught bank voles were used in the Niklasson et al. study [53]. The first colony (P-F2: N = 613) was maintained in captivity for 200 days (median) and the second colony (P-F3: N = 596) was maintained for a median of 223 days (Z = 1.034, *P* = 0.3011). Voles from both colonies were kept under 12 h artificial light conditions (0800–2000 h) and the temperature was 19±3 degrees Celsius.

Single mating pairs were established in large (“enriched”) cages following an initial observation period (to make sure the females were not already pregnant). These cages were made of transparent plastic (14.5×21.5×37.5 cm), supplied with pine shavings (Brogaarden, Gentofte, Denmark) and a wire lid with feed hopper, and a water bottle. Adding 7–8 sheets of toilet paper and two paper rolls provided enrichments. In order to prevent injuries to the pups, all breeding males were immediately removed and returned to single housing (see below) following the delivery of a litter. Breeding females were not disturbed by cage cleaning during the weaning period, unless they were PDs, in which case it could be necessary to change the cages once during the weaning period. Pups from the first colony were weaned between ages 18–52 days and all second colony pups were weaned at the age of 21 days.

When not mated in pairs, the voles were housed singly in smaller (“barren”) cages (13.5×16.0×22.5 cm) supplied only with pine shavings, a wire lid with feed hopper and a water bottle, as described in [51]. Standard pelleted rodent chow containing 19.0% crude protein, 7.0% crude ash, 6.0% crude fibres, and 4.0% crude fat (“Altromin 1324” from Altromin GmbH & Co, Lage, Germany) and water were available ad libitum. A small portion of a grain mixture containing 38.9% wheat, 22.2% maize, 22.2% peas, 11.1% red Dari (Milo) seeds and 5.6% vetch seeds (“Duefoder EX” from Brogaarden, Gentofte, Denmark) was given when the cages were cleaned (typically every two weeks, or when necessary).

### Criteria for classifying voles

According to a previous definition, voles were classified as PD if their DWI exceeded 21 ml for a minimum of one continuous month [51], [52] and onset of PD was defined as the first day the DWI exceeded 21 ml. This particular cut-off value for PD in voles stems from early work with the first colony and was originally defined by Schønecker as three times the estimated DWIs of healthy voles showing a stable DWI. Captive bank voles also frequently develop stereotypical behaviours (so-called stereotypies) in captivity, which is a type of repetitive behaviours that would probably appear strikingly abnormal and purposeless to any casual observer if exhibited in their natural habitats (for reviews of stereotypies, see e.g. [61], [62]). Approximately 30–37% of singly housed Danish bank voles are so-called stereotypers, i.e. they develop stereotypies such as backward somersaults, high-speed jumping, pacing following a fixed route or “wind screen wiper movements”, and such stereotypies will be typically exhibited in bouts, and for many hours/day [51]. Voles which neither exhibited stereotypies nor PD were classified as NN (Non-polydipsic and Non-stereotypic).

### Observations and data

DWI was calculated for all singly housed voles from weaning and until they left the colonies due to either “natural death” or “exit”. The term “natural death” is used to designate any unexplained death with no prior signs of moribundity, and “exit” from the colonies could be due to e.g. culling, transfer to other research projects, or participation in terminal experiments. The procedure involved weighing the water bottles each time a used bottle was exchanged with a new one. Data of weights and the exact time for the exchange were then entered into a database (FileMaker Pro v. 2.1 for Mac) to calculate the average 24-h water intake (DWI) in the period between replacements. Changing bottles typically took place between 1000–1200 h and DWI was calculated at least twice a week for severely PD voles and about once a week for non-PD voles. Measurements of DWI from 159 adult male PD voles showed that their average mean DWI was 46.3±11.2 ml/day, while their average maximal DWI was 63.4±19.0 ml/day (data expressed as mean/max±SD; range of valid observations/vole equals 2–72; Schønecker, unpublished data). A previous study showed that neither DWI nor onset age of PD differed between PD males and females [51].

The database also included common information such as ages at natural death/exit (and factual or presumed causes), onset ages/duration of PD and/or stereotypical behaviours, sex, periods of single/pair-wise housings, identity of partners and parents, variables related to fecundity measures, general remarks, etc. All second colony voles were weighed when weaned, and one cohort (all F1) was in addition weighed once a week until they were approx. 180 days old.

### Humane endpoints

Each vole was routinely inspected on a daily basis for signs of distress, pain, and disease. Development of what seemed to be a relatively few cases of tumours in connection to the lymphatic system (13 cases among 184 PDs) and eye problems (13 cases among 184 PDs; 2 cases among 426 non-PDs; Fischer exact *P*<0.0001) were closely followed. If it seemed as if the condition was causing pain and suffering, the vole was immediately anaesthetized by a 3∶5 CO_2_/O_2_ mixture and euthanized by cervical dislocation. The same applied to any vole showing a moribund appearance (e.g. tozzled and dull fur combined with lustreless eyes, lethargic movements, and significantly decreased attention to the surroundings). Early observations of diabetic voles showed that some would decrease their DWI considerably (e.g. from 60–70 ml to 15–25 ml) the last couple of weeks prior to their death. Accordingly, it became standard procedure to euthanize any such voles when it was clear that their water consumption showed a steady decline. Such voles would typically show additional changes in behaviour and appearance, indicating moribundity.

### Selection of data

Since not a single vole was ever kept in captivity with the prospective intention to analyse the survival following onset of PD, it was necessary to select data according to strict *a priori* criteria with the purpose of excluding known/suspected biases. In particular, two biases deserve to be mentioned: Development of stereotypies and housing effects.

The onset of PD has previously been shown to be delayed among stereotypers, as compared to non-stereotypers [51]. Furthermore, available data indicate that stereotypers experience roughly half the risk of developing PD compared to non-stereotypers, and that the risk of subsequent development of PD is roughly twice as high among pups descending from two stereotypers when compared to the risk among pups descending from two NNs (Schønecker, unpublished data).

Data from these colonies also support the observation, as first noted by Mogens Bildsøe, University of Copenhagen, that housing conditions (single vs. pair-wise housing) interact profoundly with the development of PD since both males and females, when housed in pairs, showed a markedly reduced risk of developing PD as well as a delayed onset (Schønecker, unpublished data). Housing in pairs also significantly increased survival both among stereotypers and NNs [63], and among PDs (Schønecker, unpublished data).

The following three groups (A–C) described below have been selected for further analysis and the total number of voles in these three groups amounts to 588. However, since some voles satisfied the criteria for inclusion in two, or three groups (n males/females = 38/35 and 17/11, respectively), the actual number of voles used in these three groups amounts to 459.

### Group A: Animals used to analyse survival after onset of PD

Criteria for inclusion: Born in captivity; fully outbred; singly housed from weaning until death/exit; no observed stereotypies. Data from 333 voles (155 PD: n male/female = 123/32 and 178 NN: n male/female = 73/105) were subsequently selected and analysed. The voles came from both the first and the second colony (n male/female = 128/98 and 68/39, respectively) and the survivorship of NN voles is only included for comparative reasons. Fifty-three males and 42 females from this group also satisfied criteria for use in one, or both, of the following groups.

### Group B: Animals used to analyse weight of PDs and NNs

Criteria for inclusion: Born in captivity, fully outbred, weaned at age 21 days, singly housed during all weight measurements, and no observed stereotypies during this survey. Data from 68 voles (n male/female = 38/30) were included in this survey, and their weights were measured for a maximum of 23 times. At the end of these measurements, 24 of these voles had been classified as PD (n male/female = 18/6) and 44 had been classified as NN (n male/female = 20/24). The first measurements were performed at weaning, and the following measurements (2–23) were performed once a week, and always on the same day of the week. The voles consequently differed with respect to age when weighed a given week, starting at their second measurement, where the range in age was 23–28 days (range 170–177 days at their final measurements). Voles eventually classified as PD and NN did not differ in median age when measured at the second time (25.5 vs. 27 days, respectively; Z = 1.603, *P* = 0.1089). Nineteen males and 15 females from these 68 voles were also included in the following group.

### Group C: Animals used to obtain blood glucose values

Twenty sera samples from PD voles and 40 samples from non-PD voles had been randomly drawn in 1997 from a collection of 138 available sera samples obtained in 1996–1997 from non-fasting first colony voles using cardiac puncture (equal number of male/female samples in each cohort). The samples had been analysed for blood glucose in connection with a preliminary experiment in 1997. One of the PD samples was later discharged due to uncertainty regarding the exact identity and the results from the analyses of the remaining 19 PD samples are used in this study. Two of these 19 randomly drawn samples turned out to be from PD voles, which had started to decrease their DWI shortly before sampled to a point where they were no longer showing PD. All samples came from an F2 generation (N = 59). The age (median) and weight (mean ± SD) of these 59 voles was 193 days (25/75 percentiles = 181/212 days) and 22.934±4.077 g when the sera samples were obtained. The 19 PDs had been classified as PDs for a median of 107 days (25/75 percentiles = 82.25/141) at the time of sampling, and there were no difference in age or weight between PDs and non-PDs (Z≤0.925, *P*≥0.3548 in both cases).

Other blood samples were obtained from non-fasting second colony voles in 2000 using orbital bleedings and immediately thereafter analysed for blood glucose (N = 128, of which 19 were classified as PD and two (classified as NN) showed transient PD on the day of sampling). The samples came from both wild caught adult voles (N = 67; n males/females = 29/38) and their captive born offspring (all F1; N = 61; n males/females = 34/27). Age and weight for the wild caught voles were not known with certainty at the time of the orbital bleeding procedure, whereas age and weight of their offspring was 37 days (median; 25/75 percentiles = 33/45 days) and 19.249±3.077 g (mean ± SD). The 19 PDs had at that time been classified as PD for a median of 88 days (25/75 percentiles = 38.75/101.5 days). The 59 F2 voles from the first colony were significantly older and heavier when sampled than the 61 F1 voles from the second colony (Z≥4.759; *P*<0.0001 in both cases).

We used the following data from each vole: DWI on the day of blood sampling, blood glucose value, status (PD or NN), DWI, sex, number of days since onset of PD (if PD), and finally age and weight when sampled. One single data/variable/vole was used in the subsequent calculations relating to the analysis of group C.

### Procedures for blood samplings

Blood samples were obtained from voles under 3:5 CO_2_/O_2_ anaesthesia by cardiac puncture. Before sampling, full anaesthesia was carefully ensured by the absence of normal withdrawal reflexes. The procedure lasted less than two minutes after which the vole was euthanized by cervical dislocation while still under anaesthesia. After 15 min, blood samples were centrifuged for 30 minutes at 5300 rpm and stored at −30°C until analysis. Sub-samples of 20 µl plasma were analysed (Biochemistry Analyser YSI 2700 Select), unless initial glucose levels exceeded 30 mM, in which case the analysis were repeated with 1∶4 isotonic saline solutions of sub-samples.

Other samples were obtained from un-anaesthetized voles by orbital bleeding and immediately after that analysed for blood glucose using a standard glucometer (Glucometer Elite, Bayer). Should bleeding occur after the procedure, gentle pressure was applied using a clean gauze pad against the eyeball after closure of the eyelids. All voles were monitored regularly after the procedure. According to the literature [58], [59], a casual blood glucose level exceeding 11 mM (200 mg/dl) in non-starving rodents represents clinical diabetes, just as in humans [2].

### Statistics

Data did not satisfy requirements for parametric tests. Consequently, non-parametric tests were used and data for weight and age are expressed with medians and 25/75 percentiles. The Kaplan-Meier method was used in the survival analyses followed by the Breslow-Gehan-Wilcoxon test for pair-wise differences between groups. A series of Mann-Whitney U-tests were used to analyse for weight differences between established PDs and NNs, and the Mann-Whitney U-test was also used in other pair-wise tests, when appropriate (e.g. comparing onset of PD among males and females, or lengths of captivity between the two colonies). Spearman Rank-order correlation coefficient was used to 1) correlate blood glucose values with DWI, and, 2) correlate measurements of age/weight obtained at the day of blood sampling with actual DWI. Fisher Exact was used to test for any method dependent differences in sensitivity, specificity, positive- and negative predictive values. StatView 5.0 for Macintosh was used to perform the tests, which were two-tailed, unless otherwise indicated, and corrected for continuity and ties, if necessary. Alpha was *a priori* set at 0.05.

## Results

### Survival after onset of polydipsia

**Figure 1:**
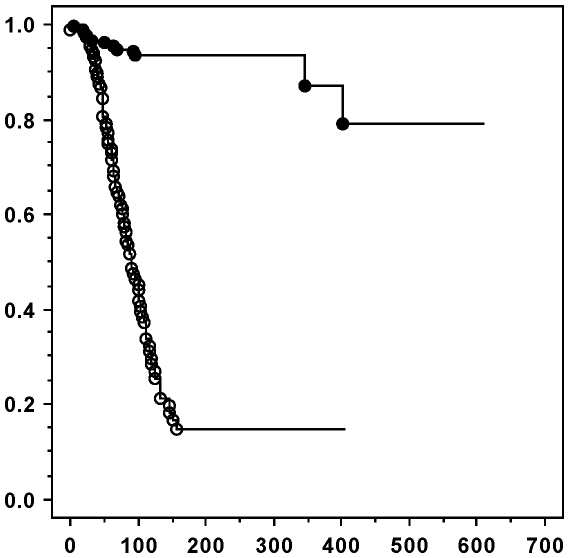
Survival following onset of polydipsia (PD). The survival of PD voles (line with open circles; n = 155) are compared with the survival of NN voles (line with filled circles; n = 178) after age 60 days. Each circle signifies an event of “natural death” (90 among PD; 13 among NN). The abscissa denotes ages in days and the ordinate denotes the fraction of survivors.

Onset of PD did not differ significantly among males and females from group A (Z = 1.484, *P* = 0.1378) and the median age at onset was 60 days (25/75 percentiles = 47/90.75 days). In order to have an unbiased reference for illustrative purposes, the survivorship of NN voles has consequently been analysed after the age of 60 days. There were no sex-related differences in survival in either PD or NN voles (Chi≤1.065; df = 1; *P*≥0.3020 in both cases), so the groups can be directly compared. If only PD voles found dead in their cages without obvious cause are considered (i.e. so-called “natural death”; n = 90), they survived their PD for a median of 67 days (25/75 percentiles = 48/101 days; maximum recording = 158 days). If the analysis is expanded to also include PDs exiting the survey for other reasons than “natural death” (i.e. so-called “Exit”; n = 65), the median survival following onset of PD was 91 days (25/75 percentiles = 57/134 days; maximum recording = 404 days). As evident in Figure 1, the data show a marked difference in survivorship between PD and NN voles (Chi = 118.297; df = 1; *P*<0.0001).

### Weight of PDs and NNs

Overall, males weighed significantly more than same-aged females after approx. one month of age, with a few exceptions (Z≥2.152, *P*≤0.0314 in all cases, except in the intervals 1, 2, 6, 9 and 10 where *P*>0.05). Consequently it would be most proper to analyse the weights of PDs and NNs using data from males and females separately.

**Figure 2:**
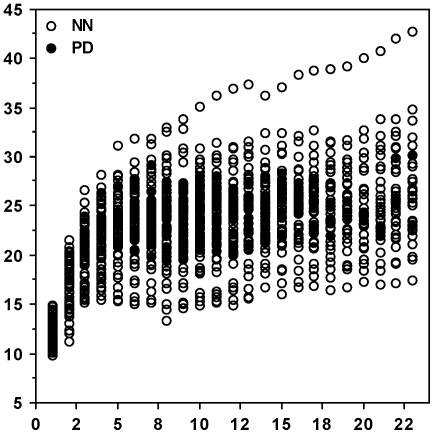
Weight of polydipsic (PD) and normodipsic (NN) voles. Weights of male and female PDs (closed circles) and NNs (open circles) through 23 weekly weight measurements at ages 21 days (session “1”) to 170–177 days (session “23”). The number of NNs through sessions 1–23 equals 68, 68, 68, 66, 62, 52, 49*, 49, 46, 46, 45, 45, 42, 39, 39, 39, 38, 36, 34, 32, 30, 29, and 29 (* one female was not weighed because of an error). The corresponding number of PDs equals 0, 0, 0, 2, 4, 14, 16, 17, 20, 20, 18, 15, 17, 15, 9, 7, 6, 4, 2, 2, 2, 2, and 2. The ordinate denotes weight (g) and the abscissa denotes the sessions.

We find that PDs and NNs do not differ significantly in weight when compared within any of the 23 intervals (males: Z≤1.878, *P*≥0.0604 in all cases; females: Z≤1.639, *P*≥0.1012 in all cases). Figure 2 shows the weight of PDs and NNs through these 23 sessions, and we find no significant weight differences between PDs and NNs (Z≤1.924, *P*≥0.0543 in all cases).

### Evaluation of polydipsia as a screening tool for hyperglycaemic voles

Both cardiac puncture (N = 59) and orbital bleeding (N = 128) were used to sample the blood for glucose determination (group C, see Table 1). When using a 21-ml/day cut-off value to diagnose PD, the sensitivities (probability of PD among voles with established hyperglycaemia) were 73% (16/(16+6); cardiac puncture) and 64% (18/(18+10); orbital bleeding). Corresponding values for the specificities (probability of non-PD among normoglycaemic voles) were 97% (36/(36+1)) and 97% (97/(97+3)), respectively. Since measures of sensitivities and specificities did not differ significantly between these methods (*P* = 0.5589 and *P*>0.9999, respectively), data could be pooled and the resultant sensitivity and specificity equalled 68% and 97% ((16+18)/(16+6+18+10) and (36+97)/(36+1+97+3), respectively).

**Table 1:** Presence/absence (+/−) of polydipsia (PD) and corresponding levels of blood glucose (BG).

	Cardiac puncture			Orbital bleedings		
	+PD	−PD	N Total	+PD	−PD	N Total
BG≥200 mg/dl	16	6	22	18	10	28
BG<200 mg/dl	1	36	37	3	97	100
N Total	17	42	**59**	21	107	**128**

Blood samples were obtained from 1^st^ colony voles by cardiac puncture (N = 59) and from 2^nd^ colony voles by orbital bleedings (N = 128).

The positive predictive values (probability that a vole is hyperglycaemic given it is PD) of the method were 94% (Cardiac puncture: 16/(16+1)) and 86% (Orbital bleedings: 18/(18+3)) and the corresponding negative predictive values (probability that a vole is normoglycaemic given it is non-PD) were 86% (Cardiac puncture: 36/(36+6)) and 91% (Orbital bleedings: 97/(97+10)). Again, data could be pooled due to the lack of inter-group differences (*P* = 0.6131 and *P* = 0.3881, respectively) and the resultant positive and negative predictive values both equalled 89% ((16+18)/(16+1+18+3) and (36+97)/(36+6+97+10), respectively).

It was possible to continue monitoring all voles subjected to orbital bleedings for subsequent developments in DWI. Thirteen voles in particular (see Table 1) did not show a positive association between a state of PD and a state of hyperglycaemia when sampled. Ten voles tested non-PD and hyperglycaemic, and of these, nine developed PD within 5–21 days following the sampling (25/50/75 percentiles = 9.3/12.0/21.0 days), whereas one individual (classified as PD seven days prior to the sampling) showed a transient decline in DWI (down to 20.9 ml/day). Three normoglycaemic voles tested PD, and of these, two showed transient PD on the day of sampling, whereas the last vole was classified as PD on the actual day of sampling due to a DWI value of 22.3 ml, increasing to 50.5 ml after a month. If both these ten non-PD hyperglycaemic voles, and the two normoglycaemic voles with transient PD, were excluded from the calculations, the resultant measures of sensitivity, specificity, positive- and negative predictive values would equal 100% (18/(18+0)), 99% (97/(97+1)), 95% (18/(18+1)) and 100% (97/(97+0)), respectively.

**Figure 3:**
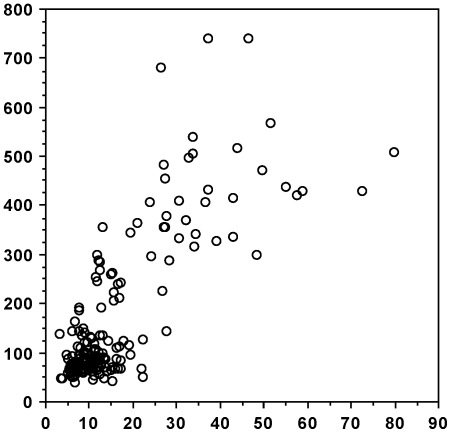
Daily water intake (DWI) correlated with levels of blood glucose (BG) – all voles. Scattergram of DWI (ml - abscissa) correlated with levels of BG (mg/dl - ordinate). Data from both polydipsic (PD) and normodipsic (NN) voles are used. N = 187.

**Figure 4:**
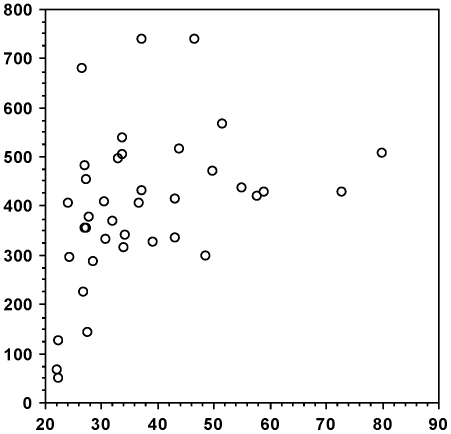
Daily water intake (DWI) correlated with levels of blood glucose (BG) – only polydipsic (PD) voles. Scattergram of DWI (ml - abscissa) correlated with levels of BG (mg/dl - ordinate). N = 38.

**Figure 5:**
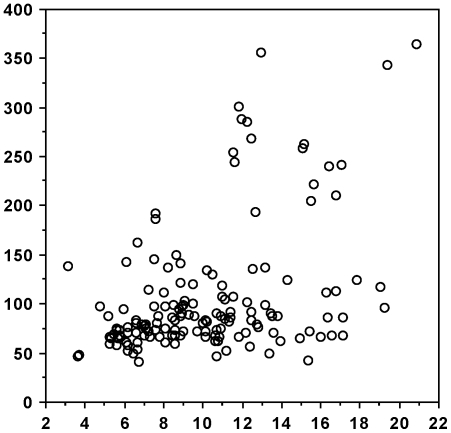
Daily water intake (DWI) correlated with levels of blood glucose (BG) – only normodipsic (NN) voles. Scattergram of DWI (ml - abscissa) correlated with levels of BG (mg/dl - ordinate). N = 149.

**Figure 6:**
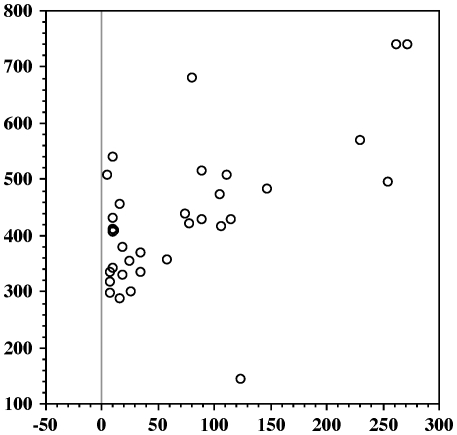
Levels of blood glucose (BG) correlated with days from onset of polydipsia (PD). Scattergram of levels of BG (mg/dl - ordinate) and days from onset of PD (abscissa). Only data from polydipsic (PD) voles are used. N = 36.

There was a positive and highly significant correlation between DWI on the day of sampling and blood glucose levels, both when all voles are considered (Figure 3: N = 187: Z = 8.645; Rho = 0.634; *P*<0.0001) and when PD voles (Figure 4: n = 38: Z = 2.964; Rho = 0.487; *P* = 0.0030) and non-PD voles (Figure 5: n = 149; Z = 4.49; Rho = 0.369; *P*<0.0001) are analysed separately. In addition, there was a significant positive correlation between blood glucose levels and time from onset of PD (Figure 6: n = 36; Z = 3.298; Rho = 0.557; *P* = 0.001). DWI also showed a positive correlation with both age (n = 101; Z = 2.376; Rho = 0.238; *P* = 0.0175) and weight (n = 101; Z = 2.645; Rho = 0.264; *P* = 0.0082) within the non-PD cohort but not in the PD cohort (n = 19; Z = 0.763; Rho = 0.180; *P* = 0.4453 (age); n = 19; Z = 1.173; Rho = 0.277; *P* = 0.2407 (weight)). Lastly, we found no sex-related differences in DWI either among the PDs (n males/females = 24/14), the non-PDs (n males/females = 68/81) or when combined (n males/females = 92/95) (Z≤1.275; *P*≥0.2024 in all cases).

## Discussion

Our present study shows that Danish bank voles have the capacity to survive for a median time of at least three months after onset of polydipsia and that survivorship for the 25% most enduring voles exceeded four and a half months with a maximum recording of 404 days. Furthermore, we have demonstrated that voles with polydipsia do not differ in weight from controls without polydipsia. Finally, we found that measurement of daily water intake to establish a state of polydipsia can be used as a tool to screen for hyperglycaemic, i.e. T1D [53], in Danish bank voles with a sensitivity and specificity of 68% and 97%, respectively, and positive/negative predictive values of both 89%.

The recorded life expectancy in diabetic Danish bank voles matches at least that of LEW.1AR1/Ztm-iddm rats which are able to survive somewhere between 4 weeks and “months” after diabetes onset [32], [46]. Bank vole survival by far exceeds the estimated two weeks survival of untreated BB-DP rats [64], the one month survival of untreated LETL rats [44], and the 1–12 weeks survival of untreated NOD mice [39], [43]. However, it must be stressed that our lowest estimate of survival following polydipsia (a median of 67 days) is solely based on the survivorship of 90 polydipsic voles, which all happened to die a “natural death” during the survey. Our highest estimate (a median survival of 91 days) includes the survivorship of a further 65 polydipsic voles which left the survey for other reasons than “natural death”. Apart from a few voles, which were killed by accidents (n = 7) or for humane reasons (n = 4), the remaining polydipsic voles would have continued to increase our estimate of possible survival, had logistical restraints or lack of scientific purpose at the time not made it necessary to terminate their stay in the colonies. Consequently both estimates must be considered conservative to a certain degree.

Polydipsia has to our knowledge only been described in studies using Danish or Swedish bank voles [48], [51]–[53], [59], [65]. However, this is more likely a matter of failing to notice what goes on in the cages rather than suggesting that the development of polydipsia among captive bank voles is an exclusively Scandinavian phenomenon. Other aspects of bank vole responses to captivity, e.g. a pronounced tendency for captive born bank voles to develop stereotypic behaviours, are indeed described among voles originating both from Denmark, Belgium, and England [48], [66], [67].

To evaluate measurements of daily water intake as a screening tool for hyperglycaemic voles, we used orbital bleeding. We cannot recommend this method to test bank voles in future experiments since bank voles have relatively small eyes compared to rats and laboratory mice and thus 8/128 of our voles experienced eye problems of varying severity subsequent to the orbital bleedings (four voles with a closed eye; one vole with a “reddish” eye; two voles with white colouration in the centre of an eye and one vole with an apparent tumour in the eye). Undesirable side effects of the method (discomfort, lesions, pain, necessity to perform euthanasia) have previously been shown to be highly dependant on the skill of the technician in studies using rats [68] and can affect subsequent behaviour [69]. Furthermore, a study using C57BL mice recently showed how samples obtained by using orbital bleedings can result in 3.5 mM higher values of blood glucose than samples obtained by using the methods of amputation of the tail tip, lateral tail incision, and puncture of the tail tip [70].

The other sampling methodology used in this study was cardiac puncture under 3∶5 CO_2_/O_2_ anaesthesia and this method has its drawbacks too. A previous study showed that humans would experience an increased feeling of anxiety, associated with increased levels of serum cortisol and ACTH (i.e. indicative of an activation of the hypothalamic–pituitary–adrenal axis), following a 15 minutes inhalation of a 35∶65 CO_2_/O_2_ mixture [71]. In partial support of this finding, a following study demonstrated significant increases in blood glucose in rats, which were fed *ad lib* and anesthetized by inhalation of 100% CO2 for a minute [72]. It is therefore conceivable that a similar mechanism could affect glycaemic measures in bank voles, and for these reasons, future researchers focussing on HPA axis activity might consider using isoflurane or sevoflurane rather than CO_2_. Especially sevoflurane has virtually no impact on blood glucose levels [73]. It might also be relevant to note that we used two different tools to analyse levels of blood glucose (the Biochemistry Analyser YSI 2700 Select and the Glucometer Elite), and whereas the YSI 2700 system has a coefficient of variation (CV) <2% [74], the Glucometer Elite has a CV of 3.7%, albeit still within the 5% CV considered acceptable for laboratory instruments [75]. However, as our results showed, we found no method-related differences in either the sensitivity, the specificity, or the positive/negative predictive values when using a state of polydipsia as a screening tool for hyperglycaemic voles.

The major advantage of using measurements of daily water intake instead of the commonly used alternatives, e.g. orbital bleeding, is that it is a quick, easy, and reasonable accurate method to provide an estimate of glycaemic status. Since the method is non-invasive and since changing water bottles is one of the standard routines in animal facilities, any subsequent effect on behaviour is not to be expected. We found the method to have a positive and negative predictive value of both 89% whereas it in theory would be expected to be closer to 100% since a state of prolonged hyperglycaemia leads to polydipsia [49], [50]). However, as mentioned in the *Materials and Methods* section, severely polydipsic and moribund voles would show a decrease in water intake a couple of weeks prior to death whereas some non-polydipsic voles might be in an early stage of diabetes when actually tested. It was indeed possible to increase all measures of accuracy to 95–100% by excluding data from voles which showed only transient polydipsia or became polydipsic within a month after the orbital bleedings, but considering the method is intended to function as a practical tool to select those individuals most likely to be hyperglycaemic, such retrospective considerations are only of academic interest.

In addition, we found a clear positive correlation between daily water intake and levels of blood glucose, and also between daily water intake and the elapsed time from onset of polydipsia. The first finding basically illustrates the physiological basis for the method to use DWI as a screening tool to pin-point voles with a high probability of also being hyperglycaemic, and the second finding indicate that an untreated T1D in voles will, just as in humans [18], worsen as time progress. Lastly, we found that whereas young non-polydipsic voles increase their daily water intake as they grow older and heavier we found no such positive correlations among polydipsic voles. The most parsimonious interpretation would be that whereas the physiological need for water increase with body size in healthy animals, the need for water in a diabetic animal is more influenced by the severity of its diabetes than of its actual age and weight. It should be noted that a relatively high daily water intake simply indicates a state of hyperglycaemia, and cannot be used to differentiate between different *types* of diabetes.

Any hypothesis proposing an infectious background for diabetes appears to have a certain innate heuristic value, and such a hypothesis has in fact been proposed, based on correlations between fluctuations in populations of wild bank voles and subsequent changes in the number of people diagnosed with T1D [76]. A virus, later named the Ljungan virus (LV), was subsequently found in saliva/lung homogenate and faeces (albeit not in the pancreas) from Swedish bank voles [77]. The study, which demonstrated T1D among wild caught Danish bank voles, also found that islets from diabetic voles stained positive for LV, suggestive of an etiological role of this member of the Picornaviridae family in combination with captivity-induced stress [53], [78]. The following study using wild caught Swedish bank voles found that the pancreas from a T1D diabetic voles stained positive for LV and clusters of picornavirus-like particles were also detected in the islets. The pancreas from an asymptomatic bank vole, however, stained negative for LV and showed no such clusters [59]. These two studies have until now been the only to demonstrate an association between LV and diabetes in bank voles, and it has often later been stated, or taken for granted, that “LV causes diabetes in voles” [79]–[84]. However, since it is not possible to determine with accuracy whether a vole is infected with the LV, it follows that its possible role as an etiological agent responsible for diabetes in bank voles remains to be proven [60]. Also the hypothesis that LV could be a zoonose relevant for human diabetes [53], [78] have found no support in subsequent independent studies [85]–[87].

Another aspect, which so far has been ignored in previous papers, is the apparent *regional differences* concerning the development of diabetes in Scandinavian bank voles. When 67 wild caught Danish bank voles were analysed after one month of captivity, 22 (33%) had developed T1D, characterized by a major loss of beta cells [53]. Also wild caught Swedish bank voles can develop a T1D, complete with a total destruction of islet cells, albeit in what appears to be a much lower fraction, since “End-stage diabetes with totally destroyed vacuolized islets was only seen in some individuals in the group kept for 8 weeks [in captivity]”, [59]. What could be a source of confusing is that some selected asymptomatic wild caught Swedish bank voles (i.e. voles which did not show signs of either polydipsia, glucosuria, hyperglycaemia or ketosis) [59], as well as 20–21% of their captive born descendants [57] would return diabetic 2-h plasma glucose values in response to a glucose tolerance test. Despite findings of islets with a “markedly changed structure containing balloon-like cells and a severe reduction in the number of endocrine cells”, the authors interpreted their results as a type 2 diabetes-like condition, or perhaps a condition resembling latent autoimmune diabetes in adults (LADA) [57]. However, it should be noted that the accompanying pictures of islets from a young diabetic Swedish bank vole (see fig. 2, middle and right panel in reference [57]) show remarkably similar features to those from an adult T1D Danish bank voles (see figs. 1c and 1d in reference [53]).

The fraction of Swedish bank voles developing overt diabetes in captivity therefore appears significantly lower than what is observed among Danish bank voles. Also the predominant type of diabetes would seem to differ, too, which might be related to a significant degree of inbreeding among the Swedish bank voles (all Danish voles studied so far have been fully outbred). The observation that a much higher fraction of Swedish wild caught bank voles tested positive for hyperglycaemia immediately after capture (21% [58] and 49% [59]), in contrast to only 4% of the Danish bank voles [53], lend further support to the notion of such apparent regional differences. Increased social stress during peak densities and declines in cyclic vole populations has been proposed to explain this particular regional difference in (presumably capture-induced) hyperglycaemia [58], [59], but given that the voles used in the Danish study [53] were caught from a population twice as dense as those reported in the Swedish studies (capture index = 6.78 vs. 3.49), other factors might be more relevant to explain the observed differences. One such factor could be simple genetic drift since Danish and Swedish bank voles have been effectively separated since the end of the last ice-age by a 4–28 km wide strait (Øresund). Recent genetic analyses have in fact also shown that Danish bank voles belong to a different phylogenetic clade than Swedish bank voles [88], so genetic differences of importance for the development of diabetes would seem to exist between Scandinavian bank voles. The rapid development of next-generation sequencing technology should make it possible to analyse the genetic background for such regional differences, even in the absence of an already fully sequenced reference genome. The principle has recently been demonstrated by Babik et al., who sequenced and *de novo* assembled the heart transcriptome from Polish bank voles in order to analyse the genetic basis for (a selection-driven) changes in metabolism [89].

In conclusion, the present study demonstrates that polydipsic Danish bank voles are capable of surviving considerable longer without insulin treatment than other T1D animal models. The majority of these polydipsic Danish bank voles are diabetic, and, according to the only relevant previous study [53], in all likelihood suffering from a T1D. This finding of exceptional ability to survive, combined with the presence of supposed diabetic long-term complications mentioned in the introduction, suggest that diabetic Danish bank voles could be useful in research addressing diabetic long-term complications in humans. The finding that polydipsic and non-polydipsic Danish bank voles weigh the same strongly suggests that they are to be considered a lean model of diabetes, more specifically of T1D. Finally, we have demonstrated an easily conducted and non-invasive method which can be used to screen for hyperglycaemic voles and could be further validated, and implemented for practical use, in other rodent models of diabetes. The development of diabetes in Scandinavian bank voles has only been the subject of a few studies and the major first obstacle to overcome will be to produce specific pathogen-free strains for laboratory use.
